# “I think it is right”: a qualitative exploration of the acceptability and desired future use of oral swab and finger-prick HIV self-tests by lay users in KwaZulu-Natal, South Africa

**DOI:** 10.1186/s13104-017-2810-7

**Published:** 2017-09-18

**Authors:** Lucia Knight, Tawanda Makusha, Jeanette Lim, Roger Peck, Miriam Taegtmeyer, Heidi van Rooyen

**Affiliations:** 1 School of Public Health, University of the Western Cape, P Bag X17, Bellville, 7535 South Africa; 2 Human and Social Development Programme, Human Sciences Research Council, 5th Floor, The Atrium, 430 Peter Mokaba Ridge, Berea, Durban, 4001 South Africa; 3 PATH, 2201 Westlake Avenue, Suite 200, Seattle, USA; 4 Department of International Public Health, Liverpool School of Tropical Medicine, Liverpool, UK

**Keywords:** HIV self-testing, Acceptability, Desired future use, Lay user perspectives, Oral swab, Finger-prick, South Africa

## Abstract

**Background:**

The uptake of HIV testing has increased in sub-Saharan Africa over the past three decades. However, the proportion of people aware of their HIV status remains lower than required to change the pandemic. HIV self-testing (HIVST) may meet this gap. Assessment of readiness for and the acceptability of HIVST by lay users in South Africa is limited. This paper presents results from a formative study designed to assess the perceived usability and acceptability of HIVST among lay users using several self-test prototypes. Fifty lay users were purposively selected from rural and peri-urban KwaZulu-Natal, South Africa. Acceptability of HIVST was assessed using a simple post-test quantitative assessment tool addressing confidence, ease-of-use, intended future use and willingness to pay. In-depth qualitative interviews explored what participants felt about the HIVST and why, their willingness to recommend and how much they would pay for a test.

**Results:**

The key finding is that there is high acceptability regardless of self-test prototype. Acceptability is framed by two domains: usability and perceived need. Perceived usability was explored through perceived ease of use, which, regardless of actual correct usage, was reported by many of the respondents. Acceptability is influenced by perceived need, expressed by many who felt that the need for the self-test to protect privacy and autonomy. Ease of access and widespread availability of the test, not at a significant cost, were also important factors. Many participants would recommend self-test use to others and also indicated that they would choose to conduct the test again if it was free while some also indicated being willing to buy a test.

**Conclusions:**

The positive response and readiness amongst lay users for an HIVST in this context prototype suggests that there would be a ready and willing market for HIVST. For scalability and sustainability usability, including access and availability that are here independent indications of acceptability, should be considered. So too should the desire for future use, as an additional factor pointing to acceptability. The results show high acceptability in all of these areas domains and a general interest in HIVST amongst lay users in a community in KwaZulu-Natal.

## Background

The UNAIDS ‘‘90 90 90’’ targets propose that 90% of people with HIV should know their status, 90% should be linked to anti-retroviral therapy (ART) and 90% should be virally suppressed by 2020 [1]. These targets require significantly increased access to, and uptake of, HIV testing that is repeated regularly [2]. South Africa’s recent HIV counselling and testing (HCT) program has yielded notable results with 13.3 million people being tested for HIV in the public health sector between 2010 and 2011 [3]. Despite large investments in facility-based HCT, the proportion of people aware of their HIV status in South Africa and sub-Saharan Africa has remained well below the levels required to positively and substantially impact the epidemic [4]. The national HIV survey of 2012 estimated that only 57% of adults had tested in the year prior to the survey and knew their status [5]. Additional HIV testing options are required to ensure early testing, linkage to care and prevention of HIV transmission-particularly of harder to reach groups such as men and young people [6].

Mobile and home-based counselling and testing models, have been shown to expand the reach of HCT to men, all women, and young people in South Africa [7–9]. Unsupervised self-administration of an HIV test or HIV self-testing (HIVST)—by an individual who then also interprets the results in private [10]—offers another alternative to standard facility-based HCT. HIVST can assist in expanding the geographic reach and uptake of HIV testing in both urban and rural locations and remove some of the barriers associated with accessing facility-based HCT [11–13].

Evidence about the feasibility and acceptability of HIVST is growing. Recent reviews on acceptability, assessed through HIVST uptake, found high acceptability across a range of both resource-poor and high-income country contexts [14], as well as within key populations [13]. The results suggest that there may be greater acceptability of oral swab testing rather than a finger-prick test [13]. Qualitative evidence from South Africa shows that lay users perceive self-tests to be acceptable but these results are based on a demonstration of an oral test and not simulation or use of a prototype [15]. In Singapore over-the-counter test kits were found to be preferable to other tests because of privacy [16, 17]. In addition, Kenyan research with health workers and their partners, and South African research with health workers, found high feasibility and acceptability of HIVST [18, 19]. Evidence for high acceptability measured both through uptake and quantitative assessment before and after supervised self-testing was also shown in a study exploring the accuracy and acceptability of the oral HIVST in Malawi [12].

Stakeholder analysis conducted as part of this research project suggest that there is a readiness and desire for the introduction of an HIV self-test in the South African context [20, 21]. While these results suggest that HIVST may have specific relevance for key populations, the authors note that the stakeholders interviewed also felt that HIVST may have general application and use [20]. Despite this evidence and self-tests being legally available in pharmacies in the country since May 2015, the most recent South African HCT policy suggests that self-testing is not recommended and further research is required, nor is there yet a policy or regulatory standards [22–24].

**Fig. 1:**
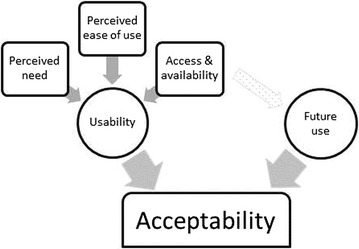
Theoretical conceptualisation of acceptability as emerging from the data

Despite the support for introducing HIVST in South Africa and some international evidence for acceptability in specific low-income contexts and situations, there are limited qualitative accounts of the acceptability of unsupervised HIVST use of prototypes by lay users themselves, particularly in the Southern African context. In this article, we use the results to frame acceptability, using two key domains, usability and desire for future use (Fig. 1). Perceived usability includes access and availability, need and ease of use. Access and availability may also affect desire for future use. Perceived usability and desire for future use independently indicate acceptability.

A prior publication addresses the usability of the prototype self-tests making use of real-time and video observations of the respondents conducting the tests to assess the respondents ability to follow the provided instructions and actually conduct the tests [25]. This research conducted in Kenya, Malawi and South Africa found that the test instructions were not always easy to follow and that errors in the practical utilisation of the prototype tests observed being conducted were common, particularly in sample collection and transfer. In a separate assessment of simulated results interpretation, the participants had difficulty interpreting results correctly. The authors recommend that to address these problems, “the ideal HIV self-test requires pictorial instructions that are clear and easy to understand, simple sample collection with integrated test components requiring fewer steps, and results that are easy to interpret” [25: S422].

This paper utilizes data from a cross-country study designed to provide feedback from Kenya, Malawi and South Africa to manufacturers during early product development regarding the target product profile [25]. This paper complements the previous research cited above by analysing data collected after the practical usability sessions were observed to explore the perceived acceptability of the HIVST prototypes among lay users from the South African arm of the study. It also provides novel preliminary qualitative evidence for policy makers considering the possible acceptability of and preferences for either oral or finger-prick HIVST in South Africa.

## Methods

The research was conducted at the Human Sciences Research Council (HSRC) offices in Vulindlela, KwaZulu-Natal, South Africa. One oral and four finger-prick HIV rapid tests and development prototypes were identified for lay user testing, based on manufacturer engagement for tests actually or near market ready [25]. Simple, context-appropriate visual and verbal test instructions were adapted based on the literature by the local research team and translated into isiZulu [26, 27]. The tests were functional and allowed for full simulation of testing, but with no regulatory approval were modified so as not to yield results. Instead, each participant was offered on-site HCT by a trained lay counsellor or a referral to a nearby clinic depending on his or her preference. The site provided a private, defined testing space for study participants.

Fifty lay users were enrolled to test the prototypes, ten for each of the five prototypes. Purposive sampling was employed to capture a diverse range of target population characteristics, such as age, sex, education (as a proxy for literacy and socio-economic status) and geographic location. Participants aged 18 years and older were recruited from the surrounding rural and peri-urban communities by a community-based mobilizer. Participants who had ever conducted an HIVST and participants who had visual impairments were excluded from the study. Ethical approval for the study was received from the national South African HSRC Research Ethics Committee. Anonymity and confidentiality of participant information was protected and a thorough written informed consent process with each participant ensured that the participants were fully aware of the potential risks and processes involved in the study.

The respondents’ perceptions of the test were collected through a simple exit interview to assess ease of and confidence with use, future use of a test if available and/or free and the respondents’ willingness to buy a test. In addition, semi-structured qualitative interviews explored these issues in further depth. Quantitative data were entered and analysed in excel using simple descriptive statistics and are used to support the qualitative findings which are the focus of this paper. The quantitative sample is not representative and therefore these results should be treated with caution and considered as indicative of perceptions further expanded on within the presentation of qualitative data and results. Trained qualitative researchers in isiZulu conducted the qualitative interviews. The interviews were audio-recorded, transcribed in isiZulu and translated into English for analysis. Initially, NVivo 10 was used to apply descriptive or topical codes [28] to the data. Codes were then reviewed and memos were created noting insights, key themes and patterns that emerged in the data on lay users perceptions of the acceptability of HIVST in South Africa.

## Results

The sample of 50 lay users included 27 women and 26 respondents from rural areas. The age range of the sample included 26 people under the age of 35 years and 39 people who had never been married. Fourteen of the respondents had completed some basic primary education, 16 some secondary and the remainder had completed high school or studied further. Four of the respondents had previously conducted another sort of home self-test. In the results respondents are only identified by gender and test prototype to ensure their identity is protected.

This section presents the results of the exit questionnaire and qualitative data together. In analysing the data for acceptability the results, suggest that there are two key domains that frame acceptability of HIVST in this context (Fig. 1): whether the self-test test is positively perceived as usable including available and accessible; and whether there is some desire or recommendation for future use. Usability is explored through perceived ease of use and perceived need; articulated in this case as overcoming barriers to the existing service. In analysing usability, it became clear that there were problems to be overcome and additional factors to be considered to ensure true usability.

### Perceived usability

Exit questionnaire quantitative data revealed that the responses to the usability of the tests were largely positive regardless of the test prototype used with over 80 per cent of respondents stating that they found the test easy to use.

*It was easy and good. Well, initially I was a bit nervous because I was just starting. But now I’m getting more comfortable because I’ve seen how it works. (male, oral swab prototype)*

The exit questionnaire data reveals that the overwhelming majority of these respondents agreed or strongly agreed with the fact that they would be confident to conduct the test again (92%). This did not differ by whether the HIVST was oral or a finger-prick test.

A number of the respondents spoke about enjoying and liking the experience of conducting the HIVST, including the ability to conduct the test alone. The privacy afforded by HIV self-testing was contrasted to the situation where someone else is present, like in a health care facility. Respondents felt that the HIVST approach was both convenient and confidential. Respondents also highlighted the way in which the HIVST bypassed other potential problems associated with facility-based HCT such as queues and filled a perceived need.

*I think it is right because sometimes there are queues at clinics. And also I am afraid that people will see me in that queue and know that I came for HIV test whereas at home it is easy and everything you do is your secret. (male, finger*-*prick prototype)*

In addition, respondents suggested that they would be likely to use a self-test in the future, if they were available for free (98%). Again, this was reported regardless of design of the prototype test. The qualitative findings support this finding and show that most of the respondents would choose to conduct the HIVST if it was available.

*I would rather not go to the clinic once I know how to use it I can then test myself. (male, finger*-*prick prototype)*

Many of the respondents mentioned the factors associated with perceived usability as those that would also encourage them to conduct the test again.

In addition to providing ways in which to bypass the problems associated with existing services and providing autonomy and privacy, the convenience associated with the test was a positive factor for almost all respondents.

#### Potential problems with HIVST use

##### Adequate delivery of counselling

Despite the overwhelmingly positive response to the HIVST, a few people in the qualitative interviews noted potential problems with the process. The most important of these was the need for counselling or basic information about the test and referrals for follow-up, as well as caution about the response of people who received a positive result from the test. For example, one respondent observed:

*When the pack arrives, I would like to have things like counselling. This should help me after the test, as to what kind of life shall I live and also understand about the test, but everything should be in the test. (female, oral swab prototype)*

Respondents were asked to provide feedback on possible sources of counselling or information about HIV that should accompany an HIV test. A number of respondents felt that counselling was essential for HIVST but that with the provision of adequate information and linkage to care this may be less of a barrier.

*…what is bad about self*-*testing is if you did not get counselling and do not have knowledge about HIV…So that will be hard…But if you know about HIV and you have been informed about it maybe you have listened to the radio or read newspapers you won’t have a problem (female, finger*-*prick prototype)*

Participants in the qualitative study suggested useful strategies for how counselling could be delivered in the HIVST package. These included the inclusion of paper-based counselling information, the sharing of information via mobile text messaging services or the use of a number to call for more information. Some respondents also suggested that the facility and clinic staff would be a means to get information and support after conducting the HIVST with also acting as a catalyst for seeking counselling and care.

*[HIVST] would be an additional thing that I have and use to test myself before going to the clinic. It’s always good going to the clinic knowing what you already have. (male, finger*-*prick prototype)*

This respondent noted that although he would like information in the test ultimately he would seek support from a health care worker. Another respondent also supported the idea of seeking health care but also reiterated the importance of having adequate information on the next steps one needs to follow testing as part of the HIV self-test kits:

*[If positive] I would go to the clinic, but even at home I would like to get counselling. I would like it to be written. (female, finger*-*prick prototype)*

It is important to note that despite these concerns about lack of counselling and the recognition that this was an important component of HIVST, with various options for delivery, respondents still felt overwhelmingly positive about the prospect of HIVST and its use for both themselves and others.

##### HIV self-testing sample collection

Qualitative interviews also provided some caveats for future HIVST use. These included ensuring that the tests were conducted correctly. Respondents also noted that the tests, particularly those requiring a finger-prick sample of whole blood, regardless of lancet type, needed better instructions and training for users. Many of these concerns related to the acceptability of using an HIV test that required finger pricking. The usability of finger-stick HIV test was problematic with many of the respondents finding the use of the lancet for whole blood collection in the finger-prick test particularly difficult, regardless of lancet type. For example some respondents called for the improvement of HIVST instructions that focused on test functioning. In addition, there were also concerns about pain and fear. For example:

*I was afraid of what I was doing…The pricking scared me…It’s painful because I would prick my finger. Then I will feel the pain. (female, finger*-*prick prototype)*

These factors may have influenced the acceptability of the finger prick HIV tests raised among the 80% of the respondents that were only offered a finger prick test and did not use an oral swab test.

#### Availability and access

Respondents expressed a desire for the HIVST to be freely available to everyone and that this should be the remit of government provided health services. Interestingly, despite reservations about testing at the clinic some respondents would be willing to access self-tests at the clinic if it meant that they would be available free.

*I would prefer to get it from the health care centre because there I get it free. I heard [the Minister of Health] say we should go to the government health care centres to get free health care attention. (male, finger*-*prick prototype)*

The results suggest the importance of getting HIVST kits free at a health care facility, but also speak to the importance of autonomy and privacy afforded to individuals if they are able to conduct the HIVST on their own, in their own space and time.

People felt that HIVST should be available for purchase at a range of places including health facilities, which were associated particularly with fee access. Respondents did make specific mention of chemists and supermarkets. Here a respondent describes a strategy for selling HIVST that she thought would work well, a strategy that was echoed by other respondents.

*This test should be sold anywhere like condoms…you find condoms everywhere even in the food store. I don’t think there should be a specific place where these test would be found… even if someone sees you going there they will know for sure that you are there to buy the test. (female, finger*-*prick prototype)*

### Future use, willingness to buy HIVST and recommendations

A desire for future use was an important part of assessing acceptability. In both the quantitative and qualitative data it was clear that in addition to using the HIVST again if it was free (98%), the majority of respondents would also be willing and likely to buy an HIVST for their personal use (86%). Notably, all participants who were neutral or not likely to buy a test again (14%) had all used a finger-prick test.

Our results indicate that respondents were willing to buy HIV self-test kits at different prices ranging from R10 to a maximum of R150, with most people willing to pay somewhere between R20 and R40. However, an outlier noted:

*I think I could pay close to R150…we normally want to get these things for free, but things that involve your life, you have to be prepared to part with your money. (male, finger*-*prick prototype)*

The likability and suggestion of high acceptability among lay users within this context was supported by evidence from the respondents on their views about the appropriateness of the HIVST for others’ use when probed on this in the qualitative interview. Many respondents felt that HIVST would be suitable for all.

Respondents were inclined to feel that HIVST would encourage greater numbers of people to test, particularly those who may be less likely to test at existing services because of barriers or factors affecting their uptake. For example, one respondent noted:

*I would recommend [HIVST] because most of the time people do not like to go to the clinic. [They] do not want to go [to the clinic] because it is far away and most people complain about the time they spend there without getting help and end up changing their minds and go back home and end up not doing what they came for…Ja they can use it… (female, finger*-*prick prototype)*

## Discussion

The paper contributes important in-depth understanding on the acceptability of unsupervised HIVST in a lay population of general users in South Africa. The results support existing research from both resource-poor and high income contexts that suggest high levels of HIVST acceptability in contexts where self-tests have been offered [11, 14]. Unlike previous research that measures acceptability purely based on uptake, this study offers an in-depth qualitative analysis of acceptability. The results of this paper use a theory of acceptability that is framed by two primary domains. Firstly, acceptability refers to respondents’ perceptions of the usability of the HIV self-tests. This is not just about comfort and confidence with the use of the self-test but also about the perceived need that the self-test fulfils. In addition, within this domain are the issues of availability and access, which are important in determining whether the self-test is perceived as usable. Secondly, acceptability is reflected in a desire or recommendation for future use of an HIVST.

Respondents report generally high-perceived ease of use of HIVST prototypes in the qualitative data and in the exit interviews through overwhelmingly positive views, including confidence with and ease of use. Literature suggests that oral swab tests may be preferable [13] to finger prick tests, particularly the analysis from the overall study [25]. However, our results show that despite concerns about potential problems with pain and reported difficulties by a minority of people, regardless of design HIVST (whether oral swab or finger-prick) self-testing was largely perceived as easy to use.

In support of findings elsewhere, respondents highlighted that HIV self-tests were more accessible and acceptable and facilitated confidentiality, privacy and most importantly removed barriers posed by facility-based services [12, 13, 24, 29–31]. This in-depth single country analysis provides concrete evidence for this in the South African context and supports the need for HIVST in order to overcome usability issues associated with traditional means of HCT.

The willingness of respondents to recommend HIVST to other people, the desire to use it again and the willingness to purchase the test were important indications of high acceptability of HIVST. This is also important because it suggests a desire to re-use a method, which may increase the frequency of use and lead to regular repeat testing, necessary to limit risk and increase prevention. It is notable that it seems that those who used the blood test may feel differently about using a test again than those who conducted the oral test, this may be related to the usability of the blood tests and the finger prick problems. A desire to use a free test was almost universal and higher than that suggested by the cross-country analysis [25], while the number of those willing to purchase the HIV self-tests was also very high. Willingness to purchase may have been linked to the desire to bypass some of the barriers associated with government provided services in order to get a more convenient service. Evidence from the literature shows that there are high rates of private (pay) health service use in South Africa, despite access to public health facilities with a very minimal charge, this suggests a desire for convenience regardless of cost [32, 33].

The findings of this study show that HIVST is acceptable to lay users within the South African context. While there are concerns with functional usability [25] these results suggest that in terms of perceptions by those who have tried to conduct tests that the tests are perceived to be usable. The results suggest that from the perspective of those included in the study, the oral test seems to be the most acceptable and the reasons for this and perceived difficulties with other tests should be further explored as policy makers consider the ideal test. The perception from respondents that the HIVST overcame many of the barriers they associate with facility-based testing is not a novel finding but it shows that HIVST may enable people who struggle with these barriers to access HIVST through an alternative route bypassing some of the challenges they perceive are associated with the facility. These results suggest that HIVST has the potential to not only reach hard to reach populations as traditionally argued within the literature such as men and young people who often don’t access facilities [13] but also those in the general population who may not access facilities for testing because of fears or negative perceptions of the service. The desire for future testing and willingness to pay for a test provide evidence for policy makers that if HIV self-testing becomes more widely available in South Africa there would be both a demand and possibly higher than expected uptake, despite reservations noted in much of the existing literature. In addition, these results suggest that repeat testing using HIVST may also increase. The caveat for policy makers about demand is that the willingness to purchase suggests that some of the barriers associated with facility-based services or other reservations about quality of freely available HIVST may influence people’s uptake of HIVST and this should be considered if plans to make HIVST available as part of government supported provision are considered.

While the potential risks associated with HIVST cannot be ignored, and require consideration, the evidence particularly from the US where HIVST has been generally available for the last few years suggest that adverse consequences may be overemphasised [34, 35]. The current WHO recommendations for the provision of HIVST suggest that it should be accompanied by adequate information and referral to relevant support, ensure the product and outcome are of good quality and regulated and that this is delivered within a rights-based framework [10]. This would be key to a properly functioning system of HIVST in South Africa.

### Limitations

The study failed to address the issue of counselling with the respondents. Respondents concerns about lack of counselling may therefore have been overstated. In addition, the study did not explore prior knowledge about HIV and testing, or testing experience and biased respondents views about need for counselling or other opinions. The qualitative sampling means that it is not possible to differentiate the findings by prototype or individual characteristics. We attempted in the analysis to make a distinction between the results by the design of the prototype but these differences should be treated with caution and would need to be tested in future larger-scale research. In addition, respondents were provided with only one test and therefore no comparison at an individual level is possible. The quantitative descriptive statistics presented as part of the analysis are indicative only of the observed perceptions of this sample and cannot be considered representative or generalizable.

## Conclusions

Access to HIVST as an alternative means of testing for both lay users and populations with traditionally low rates of HCT uptake in South Africa has the potential to contribute to increasing the proportions of the population with access to testing and therefore increase the number of people who know their status. This study provides preliminary qualitative evidence that there is high acceptability and interest in unsupervised HIVST, regardless of the design features of the test, amongst a sample of lay users in a community in KwaZulu-Natal, South Africa. This study shows that acceptability can be assessed through using two key areas; that of usability, including access and availability, and also a future desire to us a test. While there is need for larger-scale work to explore acceptability and to explore a number of additional potential problem areas such as counselling and test sensitivity, the largely positive response and readiness of these respondents, suggests that there may be a ready and willing market for unsupervised HIVST in the general population, in similar high prevalence rural and peri-urban communities in South Africa.

## Abbreviations

HIVST: HIV self-testing
ART: anti-retroviral therapy
HCT: HIV counseling and testing
HSRC: Human Sciences Research Council
